# Characterization and *in vitro* properties of oral lactobacilli in breastfed infants

**DOI:** 10.1186/1471-2180-13-193

**Published:** 2013-08-15

**Authors:** Nelly Romani Vestman, Niklas Timby, Pernilla Lif Holgerson, Christine A Kressirer, Rolf Claesson, Magnus Domellöf, Carina Öhman, Anne CR Tanner, Olle Hernell, Ingegerd Johansson

**Affiliations:** 1 Department of Odontology/Cariology section, Umeå University, Umeå, Sweden; 2 Department of Clinical Sciences/Pediatrics section, Umeå University, Umeå, Sweden; 3 Department of Microbiology, The Forsyth Institute, Cambridge, MA 02142, USA; 4 Department of Oral Medicine, Infection and Immunity, Harvard School of Dental Medicine, Harvard University, Boston, MA 02115, USA; 5 Department of Odontology/Microbiology section, Umeå University, Umeå, Sweden

**Keywords:** *Lactobacillus*, *L.gasseri*, Growth, Adhesion, Gp340, Breastfed infants

## Abstract

**Background:**

*Lactobacillus* species can contribute positively to general and oral health and are frequently acquired by breastfeeding in infancy. The present study aimed to identify oral lactobacilli in breast and formula-fed 4 month-old infants and to evaluate potential probiotic properties of the dominant *Lactobacillus* species detected. Saliva and oral swab samples were collected from 133 infants who were enrolled in a longitudinal study (n=240) examining the effect of a new infant formula on child growth and development. Saliva was cultured and *Lactobacillus* isolates were identified from 16S rRNA gene sequences. Five *L*. *gasseri* isolates that differed in 16S rRNA sequence were tested for their ability to inhibit growth of selected oral bacteria and for adhesion to oral tissues. Oral swab samples were analyzed by qPCR for *Lactobacillus gasseri*.

**Results:**

43 (32.3%) infants were breastfed and 90 (67.7%) were formula-fed with either a standard formula (43 out of 90) or formula supplemented with a milk fat globule membrane (MFGM) fraction (47 out of 90). Lactobacilli were cultured from saliva of 34.1% breastfed infants, but only in 4.7% of the standard and 9.3% of the MFGM supplemented formula-fed infants. *L*. *gasseri* was the most prevalent (88% of *Lactobacillus* positive infants) of six *Lactobacillus* species detected. *L*. *gasseri* isolates inhibited *Streptococcus mutans* binding to saliva-coated hydroxyapatite, and inhibited growth of *S*. *mutans*, *Streptococcus sobrinus*, *Actinomyces naeslundii*, *Actinomyces oris*, *Candida albicans* and *Fusobacterium nucleatum* in a concentration dependent fashion. *L*. *gasseri* isolates bound to parotid and submandibular saliva, salivary gp340 and MUC7, and purified MFGM, and adhered to epithelial cells. *L*. *gasseri* was detected by qPCR in 29.7% of the oral swabs. Breastfed infants had significantly higher mean DNA levels of *L*. *gasseri* (2.14 pg/uL) than infants fed the standard (0.363 pg/uL) or MFGM (0.697 pg/uL) formula.

**Conclusions:**

Lactobacilli colonized the oral cavity of breastfed infants significantly more frequently than formula-fed infants. The dominant *Lactobacillus* was *L*. *gasseri*, which was detected at higher levels in breastfed than formula-fed infants and displayed probiotic traits *in vitro*.

## Background

Lactobacilli colonize the normal healthy gastrointestinal tract, including the oral cavity [1]. *Lactobacillus* species have health-promoting (probiotic) traits by altering the biofilm microbial composition [2] or by stimulating the host immune response [3]. Beneficial probiotic effects come from the activity of viable organisms [4]. Probiotic action of several *Lactobacillus* species and strains has been associated with reduction of chronic inflammatory diseases [5,6] and weight regulation [7]. Lactobacilli can cause dental caries through their highly acidogenic and acid-tolerant characteristics [8], and are frequently detected in deep carious lesions [9]. Recent studies, however, suggest an additional beneficial role for oral lactobacilli [10]. Strains of *Lactobacillus paracasei*, *Lactobacillus plantarum* and *Lactobacillus rhamnosus* from caries-free subjects were found to inhibit *in vitro* growth of laboratory strains and clinical isolates of the cariogenic species *Streptococcus mutans* and *Streptococcus sobrinus* more efficiently than *Lactobacillus* strains isolated from caries-active subjects [11]. Further, in preschool children oral *Lactobacillus acidophilus* was associated with lack of caries [12].

We recently reported that lactobacilli were detected in saliva from 3 month-old breastfed but not formula-fed infants [13], and preliminary findings indicated that *Lactobacillus gasseri* was the dominant salivary *Lactobacillus*. Early colonization of cariogenic pathogens, particularly *Streptococcus mutans*, can increase the risk of childhood caries [14]. If certain *Lactobacillus* strains can suppress *S*. *mutans*, it becomes important in caries risk assessment to determine which lactobacilli are acquired in infancy and whether the colonizing strains or species have probiotic potential. Few studies, however, have examined lactobacilli in infants and probiotic activity of strains.

Breast milk provides nutrition for the infant, bacteria that can impact the microbial composition of the gastro-intestinal tract [15,16], and components that can influence bacterial attachment and growth in the mouth, stomach and intestine [17-19]. The dominant constituents in milk are lipids, lactose, oligosaccharides and proteins [20], and the major energy source in milk is triglycerides and other fats. Fats are extruded from the epithelial cell as globules that are enveloped by the epithelial cell membrane, known as the “milk fat globule membrane” (MFGM) [21]. MFGM is rich in phospholipids, gangliosides, cholesterol and many biologically active proteins [21]. The MFGM fraction participates in cellular processes and defense mechanisms in the newborn, including those involved in microbial acquisition [22,23]. MFGM proteins comprise 1-4% of the total milk protein [22], and includes seven major protein components: alpha-lactalbumin, lysozyme precursor, beta-casein, clusterin, lactotransferrin, polymeric immunoglobulin receptor precursor, and human milk fat globule EGF-factor 8 protein [23,24]. Many of these proteins are glycosylated [23]. MFGM adheres to *Lactobacillus reuteri*[25], but does not affect *L*. *acidophilus* or *L*. *gasseri*[26].

The aim of the present study was (*i*) to quantitate total lactobacilli in saliva from 4 month-old breastfed and formula-fed infants, (*ii*) to identify the dominant *Lactobacillus* species and (*iii*) evaluate possible probiotic traits of the most prevalent *Lactobacillus* species by analyzing their adhesion to host exocrine secretions and tissues (saliva, milk, purified human MFGM fraction, and epithelial cells), and their effect on growth of selected oral species *in vitro*. Here we report that oral lactobacilli are detected more frequently in breastfed than formula-fed infants, and that *L*. *gasseri*, the dominant species detected, has probiotic traits.

## Methods

### Study group

Four month-old infants were recruited from an ongoing study evaluating a novel infant formula (NCT00624689, total n=240, PI M. Domellöf, Umeå University, Sweden). Details of the parent study will be reported elsewhere (unpublished data, Timby N, Hernell O, Lönnerdal B, Domellöf M). Infants entering the parent study between September 2009 and June 2012 were invited to participate in the current study that added oral microbial sampling (saliva and oral mucosal swabs). Inclusion criteria were: 0–2 months old, birth weight 2,500-4,500 g, full term, and exclusively breast or formula-fed at the time of recruitment. The exclusion criterion was chronic illness. The parent study population aimed to recruit twice as many formula- as breastfed infants. Formula-fed infants received either a standard infant formula (Semper AB, Sundbyberg, Sweden) or an infant formula containing MFGM fraction (LACPRODAN® MFGM-10, Arla Foods Ingredients, Viby, Denmark). Infant body weight and length at birth and recruitment, vaginal or C-section delivery and use of antibiotics was obtained from medical records. Breast or bottle feeding information including type of formula given to infants before recruitment and consumption of probiotics products were obtained from infant’s diet records.

The current study population of 133 infants, comprised 43 breastfed infants, 43 standard formula-fed infants and 47 infants fed the MFGM enriched formula. Saliva could not be collected from six infants (2 breastfed, and 4 MFGM formula-fed), and oral swabs were not obtained from five infants (2 breastfed, 3 MFGM formula-fed). One standard formula-fed infant had received antibiotics at birth and one MFGM enriched formula-fed infant received antibiotics at 3 months of age. Twenty-five infants had been given commercially available probiotic oral drops (Semper Magdroppar, BioGaia AB, Lund, Sweden) containing *L*. *reuteri* ATCC 17938 (~10^8^ CFU in 5 drops) at 1, 2, 3 or 4 months of age. Infants given probiotic drops did not differ between the three feeding groups (p≥0.401).

The study was approved by the Regional Ethical Review Board in Umeå, Sweden. All caregivers signed informed consent when recruited.

### Culture of salivary lactobacilli and characterization of isolates

Whole saliva was collected from the infants and *Lactobacillus* cultured using selective medium as previously described [13]. Up to 30 isolates were selected from each plate and were identified by comparing 16S rRNA gene sequences to databases HOMD (http://www.homd.org) and NCBI (http://blast.ncbi.nlm.nih.gov/Blast.cgi).

### qPCR for *L*. *gasseri* in mucosal swabs

The mucosa of the cheeks, the tongue and alveolar ridges of the infants were swabbed using sterile cotton swabs (Applimed SA, Chatel-St-Denis, Switzerland). Samples storage, DNA purification and *L*. *gasseri* level quantification by qPCR were as described previously [13,27].

### Growth inhibition by *L*. *gasseri*

#### Cultural conditions and bacterial strains used in growth inhibition tests

*Lactobacillus* isolates were maintained on de Man, Rogosa, Sharpe Agar (MRS) (Fluka, Buchs, Switzerland) and grown in MRS broth. *S*. *mutans* strains Ingbritt, NG8, LT11 and JBP, *S*. *sobrinus* strains OMZ176 and 6715, *Actinomyces naeslundii* genospecies 1 strains ATCC 35334 and ATCC 29952, and *Actinomyces oris* (previously *A*. *naeslundii* genospecies 2) strains T14V and M4366 were maintained on Columbia agar plates (Alpha BioScience, Baltimore, Maryland, USA) supplemented with 5% horse blood (CAB) and grown in Todd-Hewitt broth (Fluka). *Fusobacterium nucleatum* strains ATCC 25586 and UJA11-a were maintained on Fastidious Anaerobe Agar (FAA, Lab M, Bury, UK) and grown in Peptone yeast extract broth (PY, Sigma-Aldrich Co., St. Louis, Missouri, USA). Bacteria were cultured anaerobically at 37°C for 48–72 h (maintenance) or 24 h (growth). *Candida albicans* strains ATCC 10231, ATCC 28366, GDH3339, GDH18 and CA1957 were maintained on Difco™ Sabouraud Maltose Agar (Becton, Dickinson and Company, Sparks, Nevada, USA) for 20 h and grown in Difco™ Sabouraud Maltose broth (Becton, Dickinson and Company) overnight under aerobic conditions at 37°C.

#### Growth inhibition by agar overlay

Five *L*. *gasseri* isolates with single nucleotide differences in the 16S rRNA gene from infants (isolate B1, B16, L10, A241 and A271) and the *L*. *gasseri* type strain CCUG 31451 (Culture Collection University Göteborg, Göteborg, Sweden) were tested for growth inhibition using an agar overlay method [11,13]. Oral bacteria tested were *S*. *mutans*, *S*. *sobrinus*, *A*. *naeslundii*, *A*. *oris* (top layers M17 agar (May and Baker, Dagenham, England), supplemented with lactose)), *F*. *nucleatum* and *C*. *albicans* (top layers same as species growth media). Agar plates without lactobacilli were negative controls. Growth was scored: 0 = no growth, complete inhibition; score 1 = moderate growth, slight inhibition; and score 2 = same or more growth as the control, no inhibition [11].

### Adhesion and aggregation tests for *L*. *gasseri*

#### Saliva, milk and MFGM fractions

Parotid saliva from two healthy adult donors and submandibular/sublingual saliva from one adult donor were collected into ice-chilled vials and used immediately or stored in aliquots at −80°C. Sterile Lashley cups were used for ductal parotid saliva collection and a custom made device for submandibular/sublingual saliva collection [28]. Breast milk from two healthy mothers was defatted [19] and stored at −80°C. Saliva and defatted milk were diluted 1:1 in adhesion buffer (ADH; 50 mM KCl, 1 mM CaCl_2_, 0.1 mM MgCl_2_, 1 mM K_2_HPO_4_, 1 mM KH_2_PO_4_, pH 7.4) and freeze-dried purified LACPRODAN® MFGM-10 diluted in ADH (1 mg/mL) were used in the experiments.

#### *L. gasseri* adhesion to host ligand coated hydroxyapatite

Following overnight culture on MRS agar, cells from *L*. *gasseri* strains B1, B16, L10, A241 and A271, and CCUG 31451 were harvested and transferred to 80 μL phosphate buffered saline (PBS: 25 mM phosphate, 85 mM NaCl, pH 7.4) with 100 μCi Trans [35^S^]-labeled-methionine (ICN Pharmaceuticals Inc., Irvine, California, USA). After overnight culture on CAB agar at 37°C in an anaerobic chamber, radiolabeled cells were harvested, washed three times in ADH buffer, and bacterial concentration determined by comparing the turbidity against a standard curve. *S*. *mutans* strain Ingbritt was cultured and radiolabeled as described [19].

Adhesion of *L*. *gasseri* to host ligands coated hydroxyapatite (HA) was performed as described [19,29]. Briefly, 5 mg HA beads (Macro-Prep Ceramic Hydroxyapatite Type II, 80 μm, Bio-Rad, Hercules, California, USA) were coated separately with human parotid saliva, submandibular/sublingual saliva, human defatted milk or LACPRODAN-MFGM-10 during end-over-end agitation for 1 h at room temperature. After washing and blocking, coated beads were incubated with radiolabeled *L*. *gasseri* (125 μl of ~1×10^9^ cells) and the bacteria were allowed to adhere for 1 h, after which the unbound bacteria were washed away. The numbers of attached lactobacilli were determined by scintillation counting.

Bacterial adhesion inhibition [19] was tested in two sets of experiments. First, *L*. *gasseri* strains were pre-incubated separately with human parotid and submandibular/sublingual saliva for 30 min at 37°C. After removal of *L*. *gasseri* cells and HA coating with pre-incubated ligand, radiolabeled *S*. *mutans* strain Ingbritt was allowed to adhere as described above. In the second set of experiments *S*. *mutans* was used for pre-incubation, and radiolabeled *L*. *gasseri* allowed to adhere for 1 h. All experiments were performed in triplicate and repeated on two separate occasions.

### *L*. *gasseri* aggregation

Equal volumes of a bacterial cell suspension (20 μL, 1×10^9^ cells/mL) with parotid, submandibular/sublingual saliva, defatted human milk or LACPRODAN® MFGM-10 (1 mg/mL) were agitated on a glass slide for 5 min at 37°C. The size of visible aggregates was rated on a scale from 0 to 4 under microscopic inspection [30].

### *L. gasseri* adhesion to human epithelial cells

The adhesive capacity of *L*. *gasseri* was examined using Human primary gingival epithelial HGEPp.05 purchased from CellnTec (CellnTec Advanced Cell Systems AG, Bern, Switzerland). Cells were cultured in CnT-24 cell culture medium (Celln Tec) at 37°C in a 5% CO_2_ incubator. The adhesion assay was performed as previously described [31]. Briefly, cells were seeded at different concentrations (0 - 10^5^ cells/cm^2^) and cultured on 4-well Lab-Tek™ II Chamber Slide™ System glass slides (Nunc, Roskilde, Denmark) at 37°C in a 5% CO_2_ incubator. Cells were then fixed in 30% acetone in methanol and the slides were blocked with 1% BSA in PBST (25 mM phosphate, 85 mM NaCl, 0,05% Tween-20, pH 7.4) for 1 h.

*L*. *gasseri* strains were cultured on MRS agar for 24 h at 37°C in an anaerobic chamber and labeled with fluorescein isothiocyanate (FITC) [32]. Lactobacilli cell density was adjusted to OD_600_ = 0.2 and stored at −80°C until use. Before addition to the gingival epithelial cell coated slides, the bacteria were diluted 4 times in 1% BSA in PBST. After incubation for 2 h, the slides were washed 300 times in PBST (buffer changed every 100 dips) and mounted for microscopy evaluation. All images were acquired using a Zeiss imager Z1 upright microscopic (Carlzeiss, Stockholm, Sweden) and software Zen 2011 with 400× optical magnification.

#### Salivary host ligands for *L. gasseri*

The presence of binding epitopes in salivary gp340 and MUC7 were evaluated by Western blot [33] for five *L*. *gasseri* isolates (B1, B16, L10, A241, A271) and strain CCUG 31451. Briefly, 0.5 × 10^8^ cells were suspended in 0.5 mL KCl buffer (50 mM KCl, 0.35 mM K_2_HPO_4_, 0.65 mM KH_2_PO_4_, 1.0 mM CaCl_2_0,1 mM MgCl_2,_ pH 6.5) and incubated under slow rotation for 1 h at room temperature with 0.5 mL parotid or submandibular/sublingual saliva diluted 1:1 in KCl buffer. Bacteria were separated from unbound salivary components by centrifugation at 13,000 rpm for 10 min at room temperature. To release the bound proteins, the bacterial pellets were boiled with 2% sodium dodecyl sulfate (SDS) for 10 min (for detection of MUC7 10 mM Dithiothreitol (DTT) was also added) and separated on 5% Tris–HCl gel (BioRad Laboratories, Hercules, Massachusetts, USA). Proteins were transferred to a (polyvinylidene difluoride (PVDF) membrane (Millipore, Bedford, Massachusetts, USA). The membranes were blocked and epitopes detected with monoclonal antibodies against gp340 (mAb143) [34] or LUM7-2 [35]. Membranes were washed with TBS (gp340) or PBS (MUC7) and incubated with HRP-conjugated anti-mouse (SAB-100, Stressgen, Victoria, Canada) for gp340 or HRP-conjugated anti-rabbit (P0448, DAKO, Glostrup, Denmark) for MUC7 and detected using Super Signal west Dura Extended Duration Substrate (Thermo Scientific, Rockford, IL, USA).

### Data processing and statistical analyses

The power calculation for the parent study was based on body weight as main outcome [36] with a statistical power of 80% and a level of significance of 0.05% (unpublished data, Timby N, Hernell O, Lönnerdal B and Domellöf M). Based on previous investigations [37], the number of infants included in this study was sufficient to detect a difference in bacterial colonization pattern.

Data handling and statistical analyses were performed using PASW Statistics 20 (IBM Corporation Route 100, Somers, New York, USA). Anthropometric measures for infants were averaged, and means with 95% CI reported. Differences between means were tested using analysis of variance (ANOVA) followed by a Bonferroni post hoc test. Differences between means for lactobacilli detected in saliva and swabs were tested using generalized linear modeling adjusted for delivery method and exposure to probiotic drops at 4 months. *L*. *gasseri* detected in swabs was additionally adjusted for amount of DNA. Categorical data are presented as proportions (%) and differences between groups were tested with a Chi^2^ test. A p-value <0.05 was considered statistically significant.

Multivariate partial least squares analysis (PLS) was performed (SIMCA P+, version 12.0, Umetrics AB, Umeå, Sweden) as previously described [38,39]. Cross-validation (Q^2^ values) was performed by a systematic prediction of 1/7^th^ of the data by the remaining 6/7^th^ of the data. The importance of each variable in the model was displayed in a loading scatter plot. R^2^- and Q^2^-values give the capacity of the x-variables to explain (R^2^) and predict (Q^2^) the outcome.

## Results

Among the 133 infants, the proportions of boys and girls, infants delivered vaginally, mean body weight and length at birth and at 4 months of age (screening age) did not differ significantly between infants fed breast milk, the standard formula or the MFGM-enriched formula (Table 1). This observation was not affected by exclusion of infants given antibiotics or probiotic drops.

**Table 1:** **Study population characteristics and *Lactobacillus *detection by feeding method**

	**Breastfed (n=43)**	**Standard formula (n=43)**	**MFGM formula (n=47)**	**p-value**
Gender (boys/girls)^1^	18/25	23/20	25/22	0.216
Vaginal delivery (% yes)^1^	95.3	88.4	83.0	0.095
Weight (gram)^2^				
At birth	3,610 (3,492-3,728)	3,481 (3,332-3,630)	3,552 (3,444-3,660)	0.352
At 4 months of age	6,742 (6,548-6,935)	6,850 (6,575-7,126)	6,859 (6,670-7,049)	0.704
Length (cm)^2^				
At birth	50.5 (50.0-51.1)	50.3 (49.7-50.9)	50.6 (50.0-51.1)	0.739
At 4 months of age	63.9 (63.3-64.5)	63.7 (62.9-64.6)	64.3 (63.7-64.9)	0.522
CFU lactobacilli/mL of saliva (log_10_)^3^	1.22 (0.20)^a,b^	0.15 (0.19)^a^	0.28 (0.19)^b^	<0.001
% (n) with lactobacilli cultured in saliva^1^				
Among all infants (n=127)	34.1% (14)^a,b^	4.7% (2)^a^	9.3% (4)^b^	<0.001
Among infants who never had antibiotics or probiotics (n=106)	33.3% (10)^a,b^	5.6% (2)^a^	11.8% (4)^b^	0.006
Among vaginally delivered infants (n=118)	35.9% (14)^a,b^	2.6% (1)^a^	8.3% (3)^b^	<0.001
% (n) infants with salivary isolates of *L*. *gasseri*^1^	29.3% (12)^a,b^	2.4% (1)^a^	7.0% (3)^b^	<0.001
*L*. *gasseri* by qPCR (pg/μL in mucosal swab samples)^4^	2.14 (0.74)^a^	0.31 (0.70)^a^	0.74 (0.68)	0.097^4^

^1^ Differences in proportions between feeding group numbers were tested with Chi^2^ test. Shared superscript letters ^(a and b)^ indicate differences between groups when tested pairwise (p≤0.008).

^2^ Data are presented as mean (95% CI) and differences between group means were tested with ANOVA.

^3^ Data are presented as mean (SE). Means are adjusted for delivery mode and exposure to probiotic drops at 4 months using generalized linear modelling (p=0.012, one sided test). Shared superscript letters ^(a and b)^ indicate groups that differ significant when tested pairwise (p-value≤0.01). The p-value between the two formula groups was p=0.439.

^4^ Data are presented as mean (SE). Means are adjusted for delivery mode, exposure to probiotic drops at 4 months (yes/no) and amount of DNA using generalized linear modelling. Shared superscript letter ^(a)^ indicates the groups that differ significantly when tested pairwise (one sided). Table 1 shows p-value between groups (p=0.097). P-values for the breastfed versus the standard formula group was p=0.040 and breastfed versus MFGM formula group p=0.089, and between the two formula groups p=0.329. 6.75×10^5^pg/mL correspond to 5.9×10^7^ CFU *L*. *gasseri* cells/mL. Employing number of bacteria/mL in the regression model leads to identical results.

### Total cultivable *Lactobacillus* in infant saliva

**Figure 1:**
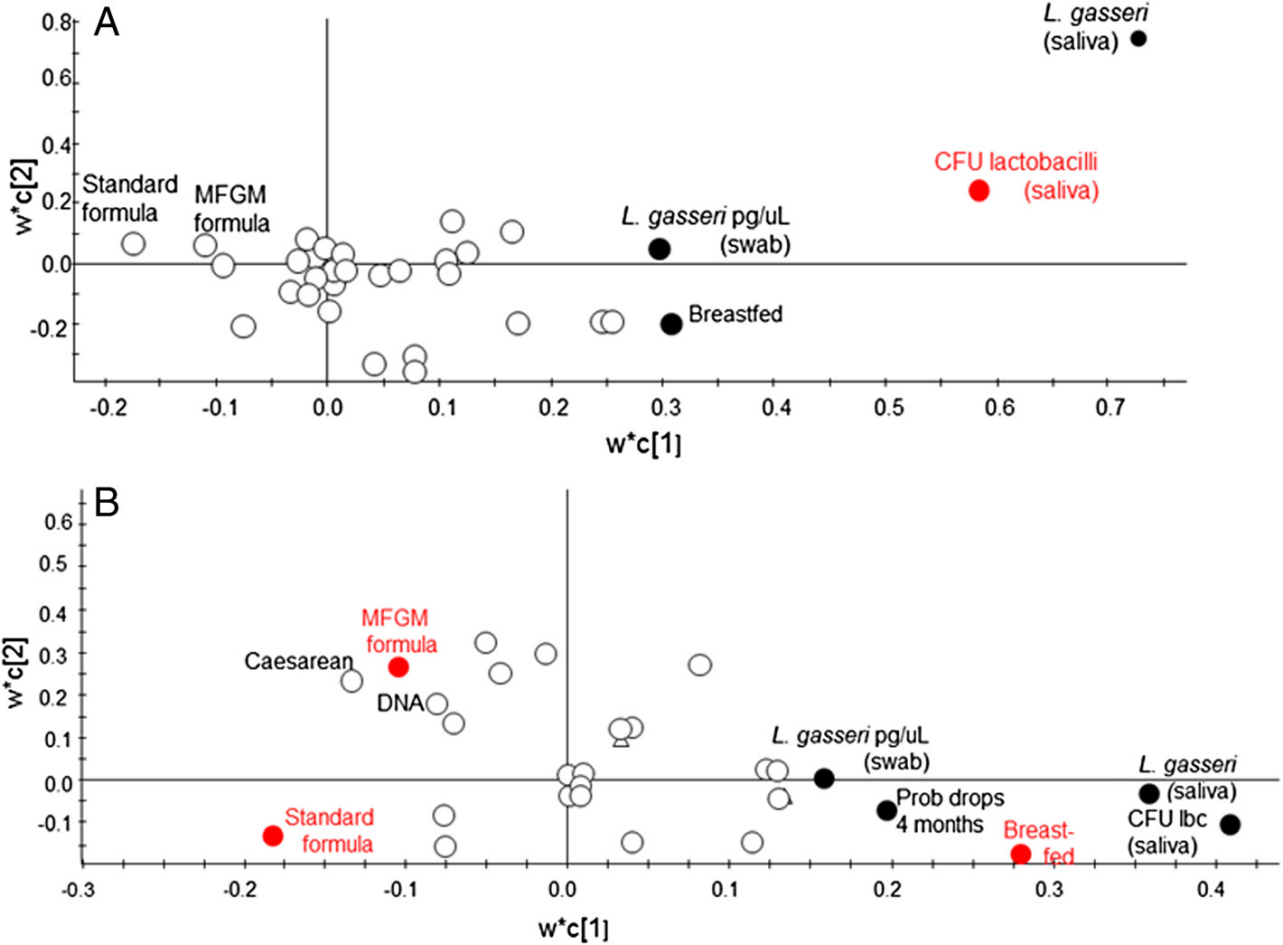
**Variable importance for *Lactobacillus *counts and feeding groups.** Partial least squares discriminant analysis identified variables influential for **(A)** Total number of *Lactobacillus*/mL saliva and **(B)** Feeding groups. Characteristics associated with the outcome variables (red circle symbol) were considered to be potential confounders and were adjusted for in statistical analysis.

Lactobacilli were cultured from saliva of 34.1% (n=14) of the breastfed infants compared with 4.7% (n=2) and 9.3% (n=4) of the standard and MFGM enriched formula-fed infants, respectively (p<0.001; Table 1). Partial least square regression (PLS) identified a feeding method (breastfeeding), *L*. *gasseri* in saliva, and *L*. *gasseri* (qPCR) in oral swabs as significantly influential for total numbers of lactobacilli/mL in saliva (dependent variable) (Figure 1A). Exposure to probiotic drops and delivery mode were positively associated with presence of lactobacilli but to a lower degree. The explanatory power of the model was 74.2% (R^2^=0.742) and the predictive power 61.4% (Q^2^=0.614). Mean CFU/mL saliva of lactobacilli (log_10_), standardized for the potential confounders probiotic drops and delivery method, were significantly higher in breastfed infants than in standard and MFGM formula-fed infants, (p≤0.001; Table 1). Presence and mean levels of salivary lactobacilli were approximately twice as high in the MFGM group than the standard formula group, but the difference was not statistically significant. Restricting the analyses to vaginally delivered infants and those who never received antibiotics and/or probiotic drops did not change findings (Table 1).

### *L*. *gasseri* in saliva and oral swabs

**Figure 2:**
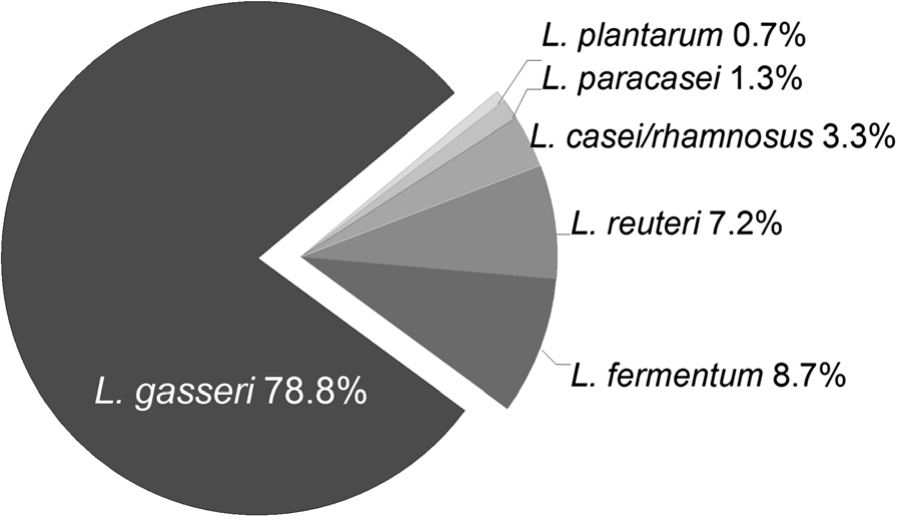
**Distribution of *Lactobacillus *species in infant saliva.** Proportions of *Lactobacillus* species in 307 isolates from MRS agar. Strains were identifed from 16S rRNA sequences.

307 putative *Lactobacillus* isolates from saliva were identified from 16S rRNA gene sequences as *L*. *gasseri* (78.8%), *Lactobacillus fermentum* (8.7%), *L*. *reuteri* (7.2%), *Lactobacillus casei*/*rhamnosus* (3.3%), *L*. *paracasei* (1.3%) and *L*. *plantarum* (0.7%) (Figure 2). *L*. *gasseri* was detected in 88% of the *Lactobacillus* positive infants. The distribution of *Lactobacillus* species detected in infants is in Table 2. Only one *Lactobacillus* species was detected in most infants (85%) (footnote Table 2).

**Table 2:** ***Lactobacillus *species isolated from 4-month- old infants**

		***Lactobacillus *species**						**Exposure to probiotics**			
		**(% of isolated colonies per infant)**^**1**^						**(age in months)**			
**Sample**	**Feeding mode**	***L. gasseri***	***L. fermentum***	***L. reuteri***	***L. casei***/ ***L. rhamnosus***	***L. paracasei***	***L. plantarum***	**1**	**2**	**3**	**4**
1	Breastfed	100								+	+
2	Breastfed	100							+	+	
3-10	Breastfed	100									
11	Breastfed	3.5	84			12.5					
12	Breastfed	3.8					96.2				
13,14	Breastfed			100					+	+	+
15	Standard formula	50			50						
16	Standard formula						100				
17-19	MFGM formula^#^	100									
20	MFGM formula^#^			100							

^1^ One species was found in 17 infants (85%), two species in two infants (samples 12, 15), and three species in one infant (sample 11).

^#^ Formula supplemented with a milk fat globule membrane fraction.

*L*. *gasseri* was detected by qPCR in 29.7% of 128 oral swabs analyzed. Generalized univariate analysis indicated that breastfed infants had significantly higher mean levels of *L*. *gasseri* in oral swabs than infants fed a standard formula (p=0.04, footnote Table 1) but not the MFGM formula. There was, however, no statistically significant difference between the three feeding groups when analyzed together (p=0.097). Means were standardized for the potential confounders of exposure to probiotic drops at 4 months, delivery mode and total DNA. In infants with cultivable salivary lactobacilli, 42.1% were positive for *L*. *gasseri* by qPCR in mucosal swabs (p=0.190), and 53.3% were *L*. *gasseri* positive by qPCR in mucosal swabs and from sequenced salivary isolates (p=0.033).

PLS modeling with feeding groups as dependent variables indicated that total *Lactobacillius* counts/mL of saliva, *L*. *gasseri* in saliva, probiotic drops at 4 month of age, and *L*. *gasseri* in oral swabs (qPCR) were influential (Figure 1B). The explanatory power of the model was 13.4% (R^2^=0.134) and the predictive power 10.3% (Q^2^=0.103).

### *L*. *gasseri* growth inhibition on oral bacteria

**Figure 3:**
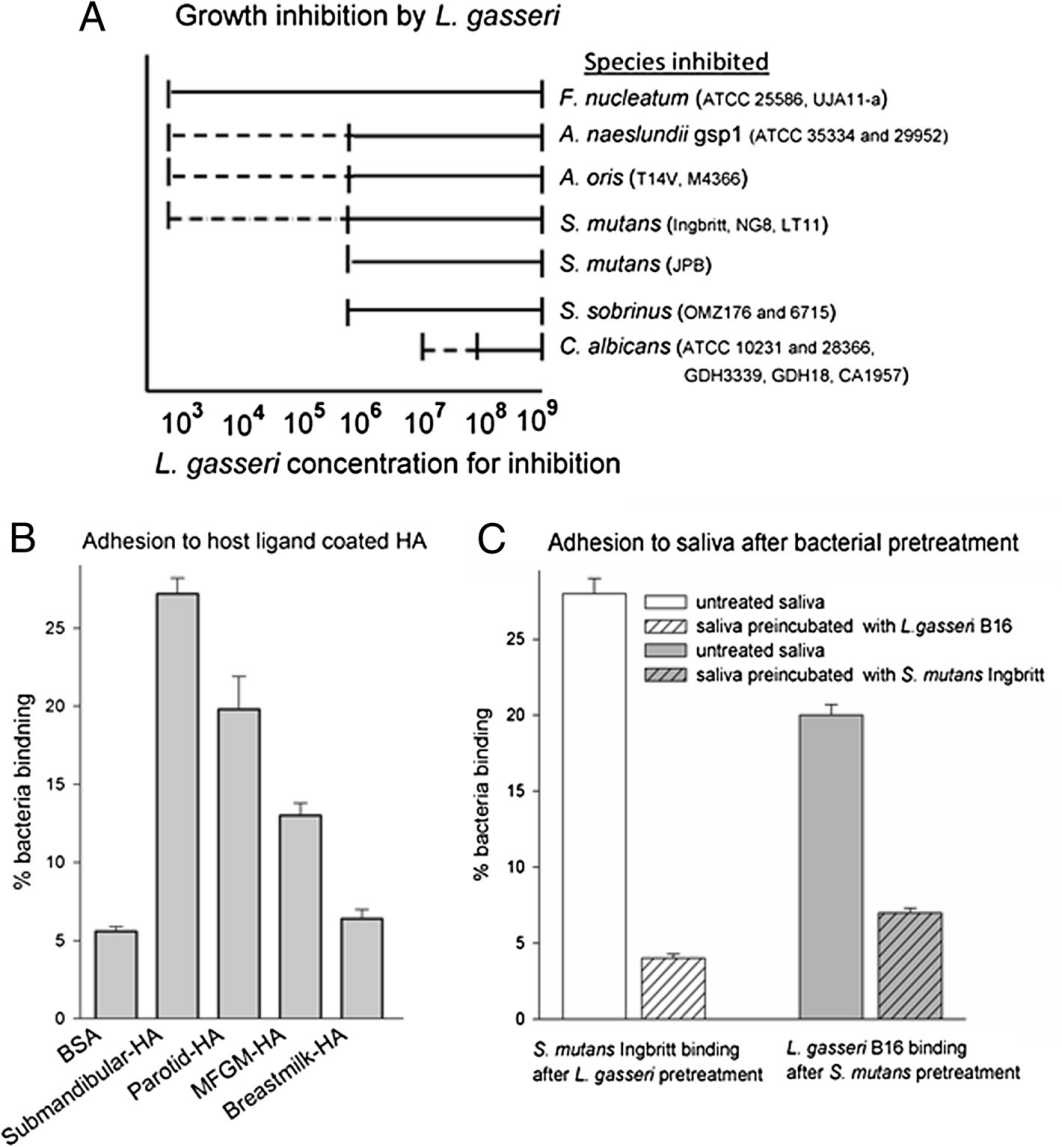
**Probiotic traits of *L. gasseri *isolates. (A)** Growth inhibition by *L*. *gasseri*. Growth of selected oral bacteria exposed to increasing concentrations of *L*. *gasseri* strain (B16) isolated from saliva. —— completely inhibited growth (score 0), - - - - - partially inhibited growth (score 1), and blank no effect on growth (score 2). **(B)** Adhesion to host ligand coated hydroxyapatite (HA). Adhesion of *L*. *gasseri* strain B16 to HA in the presence of selected host ligands. Data are presented as mean ± SEM for percent bacteria binding of added cells. Host ligands were from one adult donor of submandibular/sublingual saliva, two adult donors of parotid saliva and breast milk and purified MFGM (1 mg/mL). Background binding to bovine serum albumin blocked beads (no saliva) was <6%. **(C)** Adhesion to saliva-coated hydroxyapatite after bacterial pretreatment. Adhesion of *L*. *gasseri* strain B16 or *S*. *mutans* strain Ingbritt to parotid and submandibular/sublingual saliva before and after pre-incubation with *S*. *mutans* strain Ingbritt or *L*. *gasseri* strain B16, respectively. Data are presented as mean ± SEM for percent bacteria binding of added cells. Background binding to bovine serum albumin blocked beads (no saliva) was <6%.

Five *L*. *gasseri* isolates (B1, B16, L10, A241, A274) and the *L*. *gasseri* type strain inhibited growth of *F*. *nucleatum* strains ATCC 25586 and UJA11, *A*. *naeslundii* genospecies1 strains ATCC 35334 and ATCC 29952, *A*. *oris* (previously *A*. *naeslundii* 2) strains T14V and M4366, *S*. *mutans* strains Ingbritt, NG8, LT11 and JBP, *S*. *sobrinus* strains OMZ176 and 6715, and *C*. *albicans* strains ATCC 10231, ATCC 28366, GDH3339, GDH18 and CA1957, in a concentration dependent fashion (Figure 3A). All *L*. *gasseri* strains, inhibited *F*. *nucleatum* the most and *C*. *albicans* the least.

### *L*. *gasseri* binding to host receptors in saliva and milk

More *L*. *gasseri* B16 cells bound to hydroxyapatite coated with submandibular/sublingual saliva (27.3% cells bound) or parotid saliva (20.2% cells bound) than other strains. There was less avid binding to purified bovine MFGM fraction (13% cells bound), and binding to human milk did not exceed binding to the buffer control (Figure 3B). The binding pattern was similar for all *L*. *gasseri* strains, although the percentages of bound bacterial cells were slightly lower for four isolates than the type strain and isolate B16 (Table 3). Aggregation of *L*. *gasseri* cells by saliva showed a similar adhesion pattern to saliva-coated hydroxyapatite for all five isolates and the type strain (Table 3). Aggregation by submandibular/sublingual saliva was highest (score 3), followed by parotid saliva (score 2) and MFGM (score 2) (Table 3) and human milk (score 1) (data not shown).

**Table 3:** ***L***. ***gasseri *adhesion to saliva coated hydroxyapatite and aggregation in saliva**

	**Parotid saliva**		**Submandibular/sublingual saliva**	
***L. gasseri***	**Adhesion**^**1**^	**Aggregation**^**2**^	**Adhesion**^**1**^	**Aggregation**^**2**^
Isolate B16	++	++	+++	+++
Isolate B1	+	+	++	++
Isolate L10	+	++	++	+++
Isolate A241	+	+	++	++
Isolate A274	+	++	++	+++
Type strain 31451^T^	++	++	+++	+++

^1^ 62.5×10^6^ bacterial cells were added into each test well. + binding of <15% of added bacterial cells, ++ ≥15 to <20%, and +++ ≥20%.

^2^ – =aggregation score 0 (no visible aggregates), + aggregation score 1 (small uniform aggregates), ++ aggregation score 2 (more aggregates of slightly larger size than 1), +++ aggregation score 3 (more and slightly larger aggregates than 2) [30]. Adhesion buffer was used a negative control (score 0) and *S*. *mutans* strain Ingbritt as positive control (score +++) [18].

Adhesion of *S*. *mutans* strain Ingbritt to parotid and submandibular/sublingual saliva decreased significantly after pre-incubation of saliva with *L*. *gasseri* strain B16 (Figure 3C). A similar pattern was observed for *L*. *gasseri* binding after pre-incubation of saliva with *S*. *mutans*.

**Figure 4:**
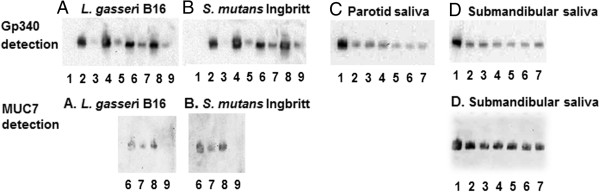
**Western blot detection of saliva gp340 and MUC7 after *L. gasseri *treatment. (A)** Upper panel shows detection of gp340 (using mAb143) and lower panel MUC7 (usig mAb LUM7-2) in parotid and submandibular/sublingual saliva alone or after incubation with *L*. *gasseri* isolate B16; **(B)** upper panel shows detection of gp340 and lower panel MUC7 in parotid and submandibular/sublingual saliva alone or after incubation with *S*. *mutans* strain Ingbritt. Numbers below lanes in panels A and B refer to the following contents: (1) Bacterial cells alone (−ve control), (2) Parotid saliva alone (+ve control), (3) Parotid saliva after bacteria incubation, (4) Bacteria incubated in parotid saliva, (5) Bacteria after SDS protein release, (6) Submandibular saliva alone (+ve control), (7) Submandibular saliva after bacteria incubation, (8) Bacteria incubated in submandibular saliva, (9) Bacteria after SDS protein release. **(C)** upper panel depicts detection of gp340 in parotid saliva alone and after incubation with five different *L*. *gasseri* isolates and the *L*. *gasseri* type strain; **(D)** upper panel depicts detection of gp340 and lower panel detection of MUC7 in submandibular/sublingual saliva alone and after incubation with five different *L*. *gasseri* isolates and the type strain. Numbers below lanes in panels C and D refer to the following contents: (1) Saliva alone (+ve control), (2) Saliva after *L*. *gasseri* CCUG31451^T^ incubation, (3) Saliva after *L*. *gasseri* isolate A241 incubation, (4) Saliva after *L*. *gasseri* isolate A274 incubation, (5) Saliva after *L*. *gasseri* isolate B1 incubation, (6) Saliva after *L*. *gasseri* isolate B16 incubation, (7) Saliva after *L*. *gasseri* isolate L10 incubation.

Gp340 (mw=340 kDa) was not detected by Western blot analysis with mAb143 antibodies in *L*. *gasseri* isolate B16 (Figure 4, upper panels A, lane 1), but gp340 was detected in parotid (Figure 4, upper panels A, lane 2) and submandibular saliva (Figure 4, upper panels A, lanes 6). The levels of gp340 were reduced in both salivas after incubation with *L*. *gasseri* (Figure 4, upper panels A, lane 3 and 7). Furthermore, bound gp340 was detected on *L*. *gasseri* (Figure 4, upper panels A, lanes 4 and 8) after incubation with saliva, and SDS treatment released gp340 bound to *L*. *gasseri* (Figure 4, upper panels A and B, lanes 5 and 9). Similar results were observed for *S*. *mutans* strain Ingbritt (Figures 4B, upper panels). The six additional isolates of *L*. *gasseri* also adhered to gp340 (Figures 4C and D, upper panels).

MUC7 (mw ≈150 kDa) was detected using Western blot analysis with mAb LUM7-2 antibodies in submandibular saliva (Figure 4, lower panels A and B, lane 6, lower panel D lane 1) but not in parotid saliva (data not shown). MUC7 levels were reduced in submandibular saliva after incubation with *L*. *gasseri* (Figure 4, lower panel A, lane 7) and *S*. *mutans* (Figure 4, lower panels B, lane 7). MUC7 was detected bound to *L*. *gasseri* (Figure 4, lower panel A, lane 8) and *S*. *mutans* (Figure 4, lower panel B, lane 8) after incubation with submandibular saliva. SDS treatment released the MUC7 bound to *L*. *gasseri* (Figure 4, lower panel A, lane 9) and to *S*. *mutans* (Figure 4, lower panels B, lane 9). Similar results were observed for MUC7 binding to six additional isolates of *L*. *gasseri* (Figure 4D, lower panel).

### *L*. *gasseri* binds to human epithelial cells

**Figure 5:**
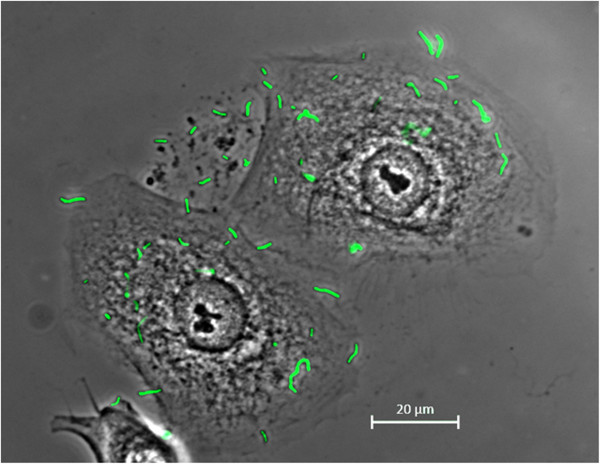
**Adhesion of *L. gasseri *to human epithelial cells.** Field of view containing differentiated human gingival epithelial cells (HGEP.05) and fluorescently stained *L*. *gasseri* A274 (in green). Bacteria were detected only in association with gingival epithelial cells. Images were captured using a Zeiss imager Z1 upright microscope. Bars in panels equal 20 μm.

Adherence of FITC-tagged *L*. *gasseri* strains was detected by fluorescence microscopy as illustrated for strain A274 (Figure 5). All *L gasseri* strains were observed only adjacent to epithelial cells.

## Discussion

In this study lactobacilli were detected more frequently in breastfed than formula-fed 4 month-old infants in saliva and mucosal swab samples as we previously observed in a different population of infants [13]. *L*. *gasseri* was the dominant *Lactobacillus* species detected, which was identified from 16S RNA gene sequences of isolates. Probiotic potential of *L*. *gasseri* was found to include growth inhibition of *F*. *nucleatum*, *A*. *naeslundii*, *A*. *oris*, *S*. *sobrinus* and *C*. *albicans* in addition to the previously reported *S*. *mutans* and *S*. *sanguinis*[13]. Other characteristics of *L*. *gasseri* were inhibition of adhesion to hydroxyapatite in the presence of saliva, salivary gp40 and MUC7 suggesting possible mechanisms for probiotic activity.

The infants sampled were recruited from a randomized clinical trial of MFGM supplemented infant formula compared with a standard formula and breastfeeding. Compliance to the feeding regimens was acceptable according to diet records obtained from the parent study. Infants recruited into the parent study were between 0 and 2 months of age. The estimated intake of breast milk at study enrollment was similar in the standard formula and the MFGM formula groups. When infants were sampled at 4 months of age, they had been exposed to either formula or breast milk for two months [40,41]. The lack of difference between the formula-fed groups suggests that this period might not have been long enough or that the different formulations do not induce changes in the oral microbiota. Previous studies, however, have observed that feeding mode, method of delivery, use of antibiotics and probiotic products may influence the oral and intestinal microbiota [2,13,40,42]. We accounted for these possible confounders in the PLS analysis, and found they had only marginally influential for feeding group allocations and total lactobacilli counts.

*L*. *gasseri* was identified as the dominant *Lactobacillus* species in the oral cavities of the 4 month-old infants. This is consistent with previous studies on *Lactobacillus* detection in the oral cavity [13,16] and the infant gut [43,44]. *L*. *gasseri* is a member of the *L*. *acidophilus* complex, which includes *L*. *acidophilus*, *Lactobacillus amylovorus*, *Lactobacillus crispatus*, *Lactobacillus gallinarum* and *Lactobacillus johnsonii*[45]. Strains belonging to the *L*. *gasseri* complex have been extensively studied for “probiotic” traits, including attachment to epithelial cells, growth inhibition, replacement or binding inhibition of pathogens and immunomodulation [46,47]. *L*. *gasseri* strains from feces and human milk have been observed to (*i*) adhere to intestinal epithelial cells and intestinal mucus (mainly MUC2) [48,49], (*ii*) produce bacteriocins [50,51], (*iii*) reduce mutagenic enzymes in feces [52], (*iv*) stimulate macrophages and lymphocytes, (*v*) modulate the immune systems through the toll receptors [53] and (*vi*) show resistance to gastric and small intestine fluids [49]. In the current report, salivary *L*. *gasseri* demonstrated several probiotic traits including: attachment to the human gingival epithelial cells HGEPp.05 and saliva, growth inhibition of several oral species and reduced attachment of the cariogenic *S*. *mutans* to saliva. Potential *in vivo* effects on the microbiota as well as short and long term biological processes remain to be demonstrated, but *in vivo* effects might be anticipated as we observed growth inhibition at *L*. *gasseri* concentrations as low as 10^3^ CFU/mL, which are the levels reported for human milk [6,16].

Studies have reported that breast milk contains *L*. *gasseri*, *L*. *salivarius* and *L*. *fermentum*, of which *L*. *gasseri* was the most prevalent species [15,16], but the prevalence of *L*. *gasseri* detection has not been reported. We cultured *Lactobacillus* species, predominantly *L*. *gasseri*, from approximately one third of breastfed infants with lower to non-detectable levels from formula-fed infants. This is consistent with our previous rapport [13]. Breast milk was not collected from the mothers, so we do not know whether detection of *L*. *gasseri* in infants reflects its presence in the mother’s milk. Other possible reasons for variability of *L*. *gasseri* detection in infants saliva include: individuality in adhesion site blocking on *L*. *gasseri* (presumably by saliva because *L*. *gasseri* aggregated in saliva but not in milk), and phenotypic host receptor variation. Few studies have examined host receptors for, and adhesion properties of, *L*. *gasseri* and lactobacilli in general [54]. Binding of various lactobacilli species to saliva gp340 [33], peroxidase [33] and gastric and intestinal mucus [46,48], blood group antigens and histone H3 [55] has been reported. Most of these host receptors are heavily glycosylated and several carry blood group antigens [55,56], which is consistent with the present findings of more avid binding of *L*. *gasseri* to submandibular/sublingual saliva, gp340, MUC7 and MFGM. Interestingly, it was reported recently [57] that the innate immunity peptide LL37, which has been detected in the mouth on epithelial cells and in submandibular/sublingual saliva [58], alters the surface of *L*. *crispatus* with a possible influence on its adhesive traits [57]. Since gp340 and MUC7 (here identified as host receptors for *L*. *gasseri* binding) exist as polymorphic variants [34,35], and phenotypic variation in gp340 relates to *S*. *mutans* adhesion avidity (gp340 here shown as shared host receptor for *L*. *gasseri* and *S*. *mutans*), it seems possible that phenotypic host receptor variation can influence *L*. *gasseri* colonization in breastfed infants. This would suggest that bacterial acquisition in infancy, and potential beneficial effects from probiotic products, may vary among individuals.

Pre-incubation of *L*. *gasseri* with saliva reduced detectable salivary gp340, and thus the observed *S*. *mutans* binding to gp340, suggesting that *L*. *gasseri* and *S*. *mutans* share a binding epitope in saliva. Competitive binding has previously been observed between *S*. *mutans* and other lactobacilli species with gp340 [33]. *L*. *gasseri* strains have also been shown to compete with, displace, and inhibit the adhesion of the enteric pathogens *Cronobacter sakazakii* and *Clostridium difficile* to intestinal mucus [48]. This suggests that *L*. *gasseri* may play a similar role in the oral cavity as has been observed in the gut. Although saliva from adults was used in the present study, gp340 has been detected in saliva in infants [19]. Saliva has been shown to have a stable pattern of salivary proteins and glycoproteins from early infancy, with the exception of albumin and the mucins, with early dominance of MUC7 later followed by MUC5B [59].

Infants fed the MFGM supplemented formula tended to have higher oral levels of total lactobacilli and *L*. *gasseri* than infants fed a standard formula. This could reflect that MFGM provides a wide range of potential carbohydrate binding epitopes on glycoproteins and glycolipids, and that *L*. *gasseri* bound to purified MFGM coated on hydroxyapatite (present study). An increased content of MFGM supplementation could potentially foster acquisition of *L*. *gasseri* and/or other *Lactobacillus* species in the gastro-intestinal tract, but this concept needs further study.

## Conclusions

Our study findings lead us to conclude that the oral cavities of breastfed infants are colonized by lactobacilli more frequently than formula-fed infants and that *L*. *gasseri* is the dominant *Lactobacillus* species. *L*. *gasseri* from infants has characteristics consistent with probiotic properties, which could influence the composition of the oral microbiota in infants.

## Competing interests

OH is member of the scientific advisory board of Semper AB.

## Authors’ contributions

IJ, MD, OH, ACRT planned, designed and financed the study. NT coordinated and organized infant participation and sampling. NRV, and PLH coordinated the oral part of the study. NRV, CÖ, CK (qPCR experiments), RC (microbiological identifications) performed laboratory experiments. NRV and IJ performed statistics and drafted the manuscript. All authors contributed to completion of the manuscript and approved it.
